# Antiplaque Effect of Essential Oils and 0.2% Chlorhexidine on an *In Situ* Model of Oral Biofilm Growth: A Randomised Clinical Trial

**DOI:** 10.1371/journal.pone.0117177

**Published:** 2015-02-17

**Authors:** Víctor Quintas, Isabel Prada-López, Nikolaos Donos, David Suárez-Quintanilla, Inmaculada Tomás

**Affiliations:** 1 Oral Sciences Research Group, School of Medicine and Dentistry, University of Santiago de Compostela, Santiago de Compostela, Spain; 2 Periodontology Unit, UCL Eastman Dental Institute, London, United Kingdom; UNC School of Dentistry, University of North Carolina-Chapel Hill, UNITED STATES

## Abstract

**Objective:**

To evaluate the *in situ* antiplaque effect after 4 days of using of 2 commercial antimicrobial agents in short term on undisturbed plaque-like biofilm.

**Trial Design and Participants:**

An observer-masked, crossover randomised clinical trial on 15 oral and systemically healthy volunteers between 20–30 years who were randomly and sequentially allocated in the same group which performed 3 interventions in different randomised sequences.

**Intervention:**

The participants wore an appliance in 3 different rinsing periods doing mouthwashes twice a day (1/0/1) with essential oils, 0.2% chlorhexidine or sterile water (negative control). At the end of each 4-day mouthwash period, samples were removed from the appliance. Posteriorly, after bacterial vital staining, samples were analysed using a Confocal Laser Scanning Microscope.

**Main Outcome Measures:**

Bacterial vitality, thickness and covering grade by the biofilm after 4 days of applying each of the mouthwashes.

**Results:**

The essential oils and the 0.2% chlorhexidine were significantly more effective than the sterile water at reducing bacterial vitality, thickness and covering grade by the biofilm. No significant differences were found between the 0.2% chlorhexidine and the essential oils at reducing the bacterial vitality (13.2% *vs*. 14.7%). However, the 0.2% chlorhexidine showed more reduction than the essential oils in thickness (6.5 μm *vs*. 10.0 μm; p<0.05) and covering grade by the biofilm (20.0% *vs*. 54.3%; p<0.001).

**Conclusion:**

The essential oils and 0.2% chlorhexidine showed a high antiplaque effect. Although the 0.2% chlorhexidine showed better results with regard to reducing the thickness and covering grade by the biofilm, both antiseptics showed a high and similar antibacterial activity.

**Clinical Relevance:**

Daily essential oils or 0.2% chlorhexidine mouthwashes are effective when reducing dental plaque formation in the short term. Although 0.2% chlorhexidine continues to be the “gold standard” in terms of antiplaque effect, essential oils could be considered a reliable alternative.

**Trial Registration:**

ClinicalTrials.gov NCT02124655

## Introduction

The accumulation and maturation of oral biofilm in the gingival margin is widely recognised to be the primary aetiological factor in the development of chronic gingivitis [1,2]. Based on this association, the current treatment of gingivitis is focused on biofilm disruption, which will normally include mechanical processes, both professionally and at home. However, for patients, it is not easy to achieve a proper level of plaque control. The efficient plaque control techniques are very time consuming and require a special motivation and skills for their optimum use [3]. It was at this point where mouthwashes become important, due to the fact that they include diverse types of antimicrobial agents to complement the results of mechanical oral hygiene measures [4].

Chlorhexidine (CHX) is considered the “gold standard” of oral antiseptics; nevertheless it has not been recommended for long periods of time due to its well-known secondary effects [5]. All of these inconveniences have limited its acceptability among dental professionals and users; in contrast, however, are the exceptional antiseptic properties, promoting the interest of researchers in other alternative antiplaque agents [6]. Mouthwashes containing essential oils (EO) in their formulation have received a lot of attention. Their antiplaque activity has been demonstrated in numerous clinical studies, in which they were used in conjunction with mechanical oral hygiene measures [7,8].

Taking into account the fact that bacteria present in a biofilm can be from 10–1000 times more tolerant to antimicrobial agents than those in planktonic phase [9], a more predictive evaluation of the efficacy of an antiseptic agent present in a mouthwash could be done using biofilm models. Numerous studies have been carried out regarding the activity of EO and CHX on the oral biofilm both *in vitro* and *in situ*. The latter are more valuable when establishing the antiseptic efficacy of mouthwashes, due to the fact that their activity is tested under *in vivo* clinical conditions [10].

In order to achieve a better understanding of the clinical effects that these agents produce in the interior of the biofilm, it is necessary to apply a methodology in which the biofilm grows directly in the interior of the oral cavity but its three-dimensional structure is not distorted by manipulation [11,12]. Most *in situ* studies on “non-destructured” dental plaque do not use natural teeth; instead, they use disks made of different materials that are introduced in the mouth for a variable period of time, during which they are exposed to the intraoral conditions of an individual. Therefore, it is not dental plaque itself, but a biofilm which is presumably very similar to dental plaque, which is generated in similar conditions and is set on a substrate which is generally artificial. Hereinafter, it will be denominated plaque-like biofilm (PL-Biofilm) [13,14].

The study of the antiplaque effect of an antimicrobial agent can be performed in long and short term clinical studies. Among the latter, 4-day models have particular importance. This model can be described as an established method for assessing the inhibitory activity against dental plaque that mouthwashes have, *per se*, and determines the relative effectiveness of the different formulations [15,16]. Thereby, they have been widely used by different research groups to study various antiplaque agents that are commonly used in the oral cavity [15–19].

Confocal laser scanning microscopy (CLSM), despite having poorer resolution than transmission electron microscopy (TEM) or scanning electron microscopy (SEM) [20], has eliminated or considerably reduced the distortion produced by preparation of the samples. The main advantage of CLSM is that it permits the analysis of biofilm *in situ*, without altering its delicate structure, keeping it hydrated with no need for fixation or drying [21,22]. CLSM also facilitates the observation of biofilms *in situ* and in “real” time, with all of the benefits of most sophisticated image analysis [23].

Systems based on the identification of bacterial vitality by fluorescence have become increasingly important, since they are accepted as a simple, accurate, reproducible and highly sensitive method for the quantitation of attached microorganisms [23]. Consequently, numerous authors have examined biofilms with the help of CLSM and fluorescence staining, incorporating both to analyse their structure [11,24], such as the spatial distribution of the vital and non-vital bacteria [13,25,26]. Various combinations of dyes have been used in the literature [10,11,27–29]. The combination of SYTO 9 and propidium iodide (PI) has been posed as a reliable alternative [30] to traditional blend of fluorescein diacetate with ethidium bromide, mainly because of the destructive properties of this combination and the toxicity and instability of the ethidium bromide [30]. This staining method allows for a visual demonstration of bactericidal activity as well as its quantification using computerized image analysis [31].

The aim of this study was to evaluate the *in situ* antiplaque effect of 2 antimicrobial agents in the short term with a posterior analysis on “non-destructured” biofilm with CLSM combined with fluorescence staining.

## Materials and Methods

This was a randomised, observer-masked, crossover study of the antiplaque efficacy of 2 available formulas based on EO (Listerine Mentol Listerine Johnson & Johnson, Madrid, Spain) and 0.2% CHX (Oraldine Perio Johnson & Johnson, Madrid, Spain) on an *in situ* model of PL-Biofilm growth. The protocol for this trial and supporting CONSORT checklist are available as supporting information; see S1 CONSORT Checklist and S1 Protocol. The trial was registered in ClinicalTrials.gov with the ID number NCT02124655. The authors confirm that all on-going and related trials for this intervention are registered. URL of the registered trial: (http://clinicaltrials.gov/ct2/show/NCT02124655?term=essential+oils+chlorhexidine+biofilm&rank=1).

### Selection of the study group

**Fig 1:**
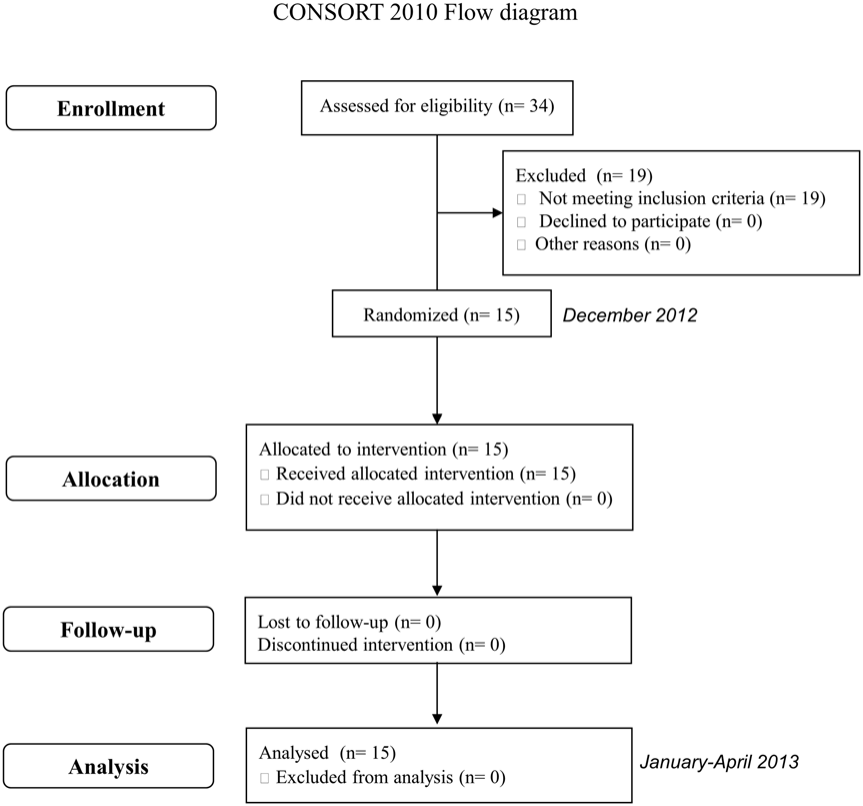
Flow diagram of the study with enrollment, allocation, follow-up and analysis of participants.

To calculate “a priori” sample size, the following statistical criteria were established: an effect size of 0.35, an alpha error of 0.05 and a statistical power of 80%. Assuming these criteria and the possible application of repeated measures ANOVA test, a sample size of 15 subjects was required (Fig. 1). The sample size calculation was performed using the program G*Power 3.1.5. The participants were collected among dental students at the Faculty of Medicine and Dentistry of Santiago de Compostela where volunteer enrollment was asked by advertisements asking for the participation in a research study at the Faculty hall. All of these volunteers were revised by the same calibrated clinician to ensure they fulfilled all inclusion and exclusion criteria. The volunteers chosen were systemically healthy adult volunteers between 20 and 45 years old, who presented a good oral health status: a minimum of 24 permanent teeth with no evidence of gingivitis or periodontitis (Community Periodontal Index score = 0) [32] and an absence of untreated caries at the beginning of the study. The following exclusion criteria were applied: smoker or former smoker, presence of dental prostheses or orthodontic devices, antibiotic treatment or routine use of oral antiseptics in the previous 3 months, and presence of any systemic disease that could alter the production or composition of saliva. Before the start of each study, a full mouth scaling with ultrasonics and teeth polishing with rubber cup after dental disclosing was performed by the same calibrated clinician on all selected participants.

This project was approved (number 2012/393) by the Clinical Research Ethics Committee of Galicia. Written informed consent was obtained from all participants in the study.

### Production of the Intraoral Device of Overlaid Disk-holding Splints (IDODS)

After considering a number of previously described *in situ* models [10,24,25,33], an individualised splint of the lower arch was created for each volunteer, which was capable of holding 6 glass disks (6 mm in diameter, 1 mm thickness); these were polished at 800 grit. The characteristics of this splint have been previously described [34], although a new variety of partial splint has been already introduced [14].

### Mouthwash protocol

During the 96 hour (4 days) duration of each course of study, each volunteer wore the splints with the glass disks, withdrawing them from the oral cavity only during meals and to perform oral hygiene procedures, using only the mechanical removal of bacterial plaque with water, without the use of any toothpaste or mouthwash. They were advised to do just 3 meals per day avoiding eating or drinking in-between. The only drink permitted during meals was still water. While eating and brushing their teeth, the splints were stored in a provided opaque plastic box (the type used to store orthodontic removable devices). Volunteers were told to impregnate a sterile gauze with 5 ml of saline (the gauzes and the saline were provided as well), and extend it on the base of the plastic box, support the splints on it and close the box, leaving it at room temperature. The maximum allowed time that the volunteers had to eat and perform the oral hygiene measures were 20 minutes.

Using the permitted mechanical oral hygiene measures (without the splints), the volunteers performed the following protocols based on the manufacturers’ instructions, with the splints in the oral cavity, during the 4 days in the morning (8.30) after breakfast and at night (22.00) after dinner:

A) 20 ml rinses for 30 seconds with essential oils/twice daily (4D-EO).

-or-

B) 10 ml rinses for 30 seconds with 0.2% chlorhexidine/twice daily (4D-0.2% CHX; positive control).

-or-

C) 20 ml rinses for 30 seconds with sterile water (4D-WATER; negative control).

**Fig 2:**
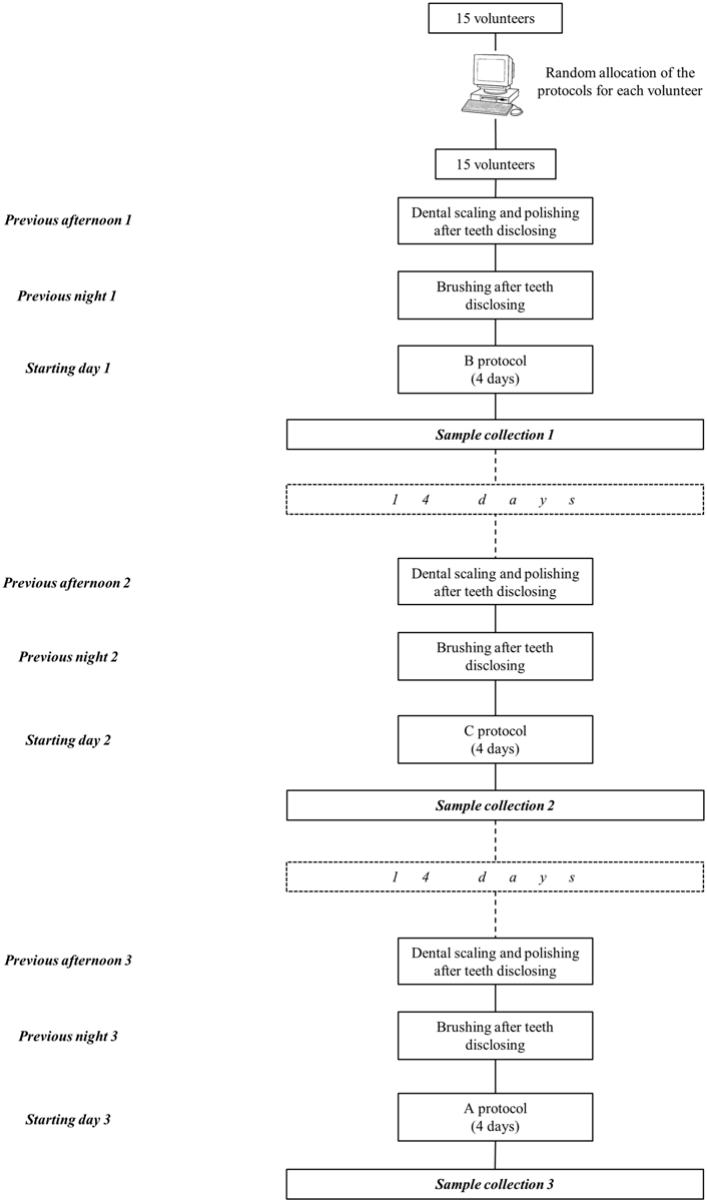
Scheme of the sequence of protocols followed by the volunteers.

A number was assigned to each participant (closed envelope) by an investigator who was unaware of the study design. Using an internet-based balanced randomisation system [35] introducing the numbers and the 3 test cycles (A, B and C), a random sequence indicating the mouthwash that each subject would use first, second and third was obtained. Although they were not told the type of mouthwash they were going to use, the obvious differences in taste between the 3 mouthwashes made allocation concealment to the volunteer impossible. The antiseptics/control were prepared in opaque bottles labelled with an A, B, or C depending on the containing solution, with 10 ml more than the quantity needed for completing the whole series of mouthwashes. The day before the start of each experiment, after the full mouth dental scaling and polishing, participants were given by a masked investigator the corresponding previously made splints, the allocated opaque bottle, a plastic glass and a sterile 20 ml serum syringe with the objective of being the more precise possible with the quantity of solution used for the mouthwash. The day of the sample analysis, they were asked to bring back the bottles to measure the quantity of solution left in the bottle. All volunteers performed the 3 rinsing cycles, with a rest period of 2 weeks between each test (Fig. 2).

### Study endpoints

The primary endpoints with respect to the antiplaque efficacy of the 2 antiseptics on the PL-Biofilm were the bacterial vitality, in terms of detection of non-vital bacteria in the biofilm; the thickness and the covering grade, in terms of quantification of height and density of biofilm, respectively.

### Collection of the samples of PL-Biofilm

Sample collection was done individually at the Unit of Confocal Microscopy of the University of Santiago de Compostela at 8 am in the morning, so that the samples of each volunteer were analysed on different days. It was determined that a minimum of 10 hours should have elapsed since the last mouthwash on the previous night.

As the glass disks (in total, 6) were removed from the splint, they were immediately immersed in 100 μL of fluorescence solution LIVE/DEAD BacLigh and kept in a dark chamber at room temperature for 15 minutes. The characteristics of LIVE/DEAD BacLight fluorescence solution (Molecular Probes, Leiden, the Netherlands), as well as its preparation, have been described by the authors in a previous paper [36]. Microscopic observation was performed by a single investigator who was unaware of the study design, using a Leica TCS SP2 laser scanning spectral confocal microscope (Leica Microsystems Heidelberg GmbH, Mannheim, Germany) with an HCX APOL 63x/0.9 water-immersion lens.

### Processing of the samples of PL-Biofilm

**Fig 3:**
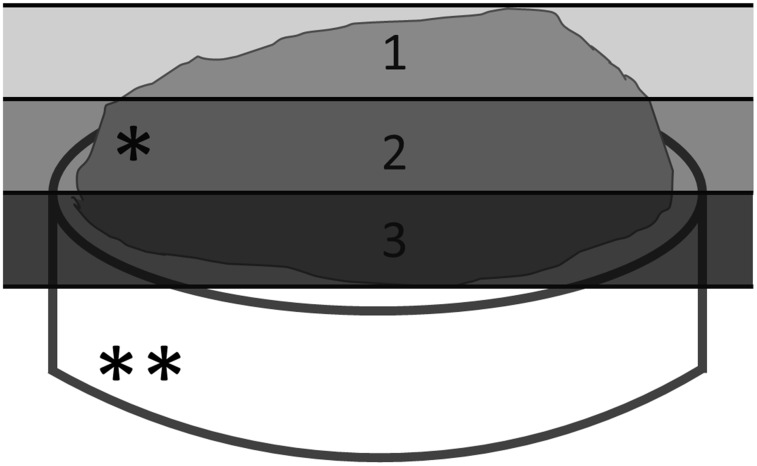
Scheme of the divisions of the PL-Biofilm in layers. 1-Outer layer; 2-Middle layer; 3-Inner layer. *PL-Biofilm. **Glass disk (substrate).

Four selected fields or XYZ series in the central part of each disk were evaluated. These fields were considered representative of the whole sample after the observer’s general examination. Fluorescence emission was determined in a series of XY images in which each image corresponded to each of the Z positions (depth). The optical sections were scanned in 1 μm sections from the surface of the biofilm to its base, measuring the maximum thickness of the field and subsequently the mean thickness of the biofilm of the corresponding sample. The maximum thickness of biofilm field was defined as the distance between the substrate (in perpendicular) and the peaks of the highest cell clusters [37]. The maximum biofilm thickness of each field was divided into 3 zones or equivalent layers: outer layer (layer 1), middle layer (layer 2) and inner layer (layer 3) (Fig. 3).

The capture of the data was done with the same settings in all cases. The spatial scan mode (XYZ) and the 1024x1024 pixels scan format resolution were used. The Argon-ion and DPSS laser were used at a 13% and 78% of maximum intensity, respectively. The values for the pinhole, zoom and scan speed were 121.58 microns, 1 and 400Hz, respectively. The only values that were different depending on the sample were the offset (range between-1% to 1%) and PMT gain which was different for channel red and green, being in general terms, higher for green than for red (test and positive control), due to the fact that there was more presence of red than green signal, being for the negative control the opposite. These values were always adjusted to get a good quality capture without background noise, avoiding excessive saturation of the brightest pixels of the image. As the technician was blinded to the experiment, they were advised to make the adjustments always consistent with what was seeing by the objective of the microscope, obtaining an image which was the closest as possible to reality.

Quantification of bacterial vitality in the series of XY images was determined using cytofluorographic analysis (Leica Confocal Software). In this analysis, the images of each fluorochrome were defined as “channels” (SYTO 9 occupies the green channel and PI the red channel). Square capture masks were used to measure the area occupied (μm^2^) by the pixels in each channel, determining the total area occupied by the biofilm and the corresponding percentage of vitality. The intensity ranges that were considered as positive signal were between 100 and 255. Determination of the mean percentage of bacterial vitality in each field required sections with a minimum area of biofilm of 250 μm^2^, and the mean percentage of bacterial vitality of the biofilm was calculated for the corresponding sample and for each biofilm layer.

For quantification of the percentage of surface substrate covered by the biofilm (covering grade), the cytofluorogram itself was used. From the maximum projection (superposition of all planes captured) of each of the analysed fields, the percentage of covering grade was obtained by calculating the sum of the bacterial mass (vital and non-vital) in regard to the total surface of the field (% positive within total area).

### Statistical analysis

The results were analysed using the PASW Statistics Base 20 package for Windows (IBM, Madrid, Spain) by an investigator who was blinded to the type of interventions analysed. All values from the quantitative variables analysed (“biofilm thickness” and “bacterial vitality percentage”) presented a normal distribution, which was determined using the Kolmogorov-Smirnov test. One-way ANOVA with repeated measures was used for intra-mouthwash (differentiating between the 3 biofilm layers) comparisons using all of the PL-Biofilm samples. Two-way ANOVA with repeated measures was used for inter-mouthwash (differentiating between the 3 biofilm layers) comparisons using all of the PL-Biofilm samples. Pairwise comparisons (with the Bonferroni adjustment) were used for the analysis of intra- and inter-mouthwash results (including differentiating between the 3 biofilm layers). Statistical significance was taken as a *p* value less than 0.05.

## Results

A total of 34 volunteers were evaluated for eligibility to achieve the calculated sample size (n = 15). When this number of participants meeting the inclusion and exclusion criteria was reached, the enrollment process was stopped. A total of 19 subjects were not selected as eligible for not fulfilling the inclusion criteria. In relation to demographic characteristics of the selected participants, 8 were female and 7 male with a mean age of 25.4 ± 2.3 years. No adverse or side effects were observed by investigators or reported by the volunteers after the completion of any of the rising cycles.

### PL-Biofilm: thickness, bacterial vitality and covering grade after 4-day-rinsing periods with essential oils and 0.2% chlorhexidine

**Fig 4:**
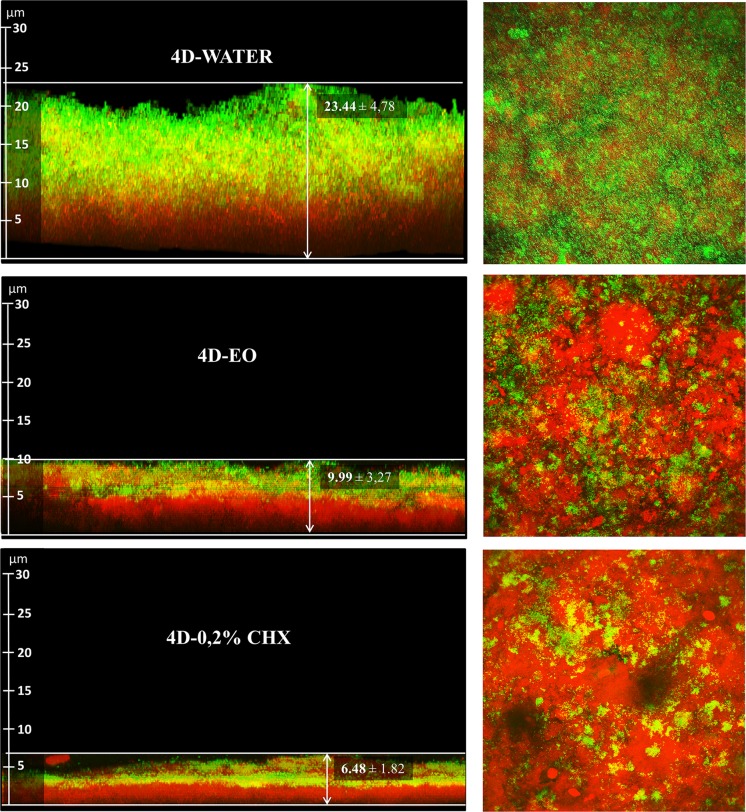
On the left, lateral projections of the brightest point (X-Z) from images stacked in Y plane from the three rinsing cycles, presenting their respective mean thickness. On the right, maximum projections of the brightest point (X-Y) from images stacked in Z plane. 4D-WATER = 4-day period during which rinses with 20 mL of sterile water are done twice a day; 4D-EO = 4-day period during which rinses with 20 mL of essential oils are done twice a day; 4D-0.2% CHX = 4-day period during which rinses with 10 mL of 0.2% chlorhexidine are done twice a day.

**Fig 5:**
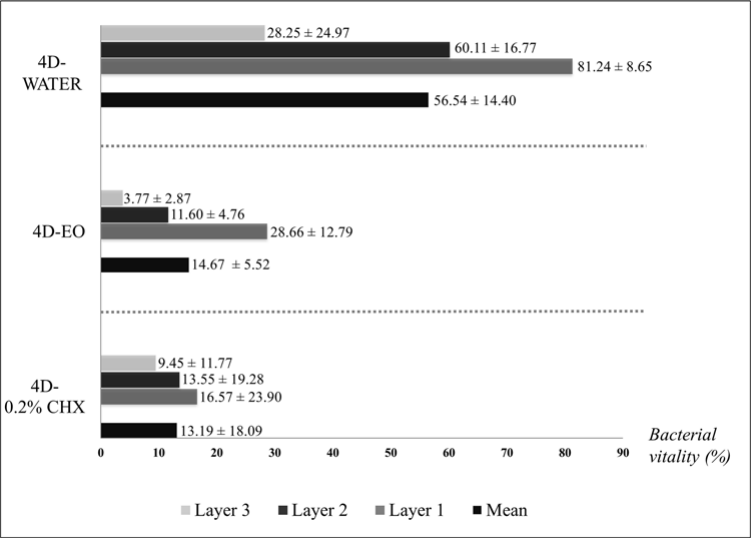
Bacterial vitality in percentages. Total and different layers in each rinsing cycle. 4D-WATER = 4-day period during which rinses with 20 mL of sterile water are done twice a day; 4D-EO = 4-day period during which rinses with 20 mL of essential oils are done twice a day; 4D-0.2% CHX = 4-day period during which rinses with 10 mL of 0.2% chlorhexidine are done twice a day. Layer 1 = outer layer; Layer 2 = middle layer; Layer 3 = inner layer.

**Fig 6:**
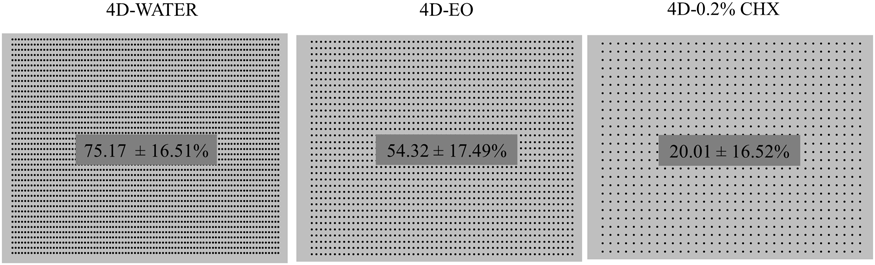
Scheme of the area occupied by the PL-Biofilm after the different rinsing cycles. Presentation of the obtained cyphers. 4D-WATER = 4-day period during which rinses with 20 mL of sterile water are done twice a day; 4D-EO = 4-day period during which rinses with 20 mL of essential oils are done twice a day; 4D-0.2% CHX = 4-day period during which rinses with 10 mL of 0.2% chlorhexidine are done twice a day.

Both 4D-EO and 4D-0.2% CHX protocols were found to be significantly effective in regard to the 4D-WATER for reducing the thickness (9.99 ± 3.27 μm and 6.48 ± 1.82 μm respectively *vs*. 23.44 ± 4.78 μm) (Fig. 4) (S1, S2 and S3 Tables) and the bacterial vitality (14.67 ± 5.54% and 13.19 ± 18.09% respectively *vs*. 56.53 ± 14.40%) (Fig. 5) (S4 Table [see A], S5 Table [see A] and S6 Table [see A]). The covering grade was also significantly reduced by the 4D-EO and 4D-0.2% CHX in regard to the 4D-WATER (54.32 ± 17.49% and 20.01 ± 16.52% respectively *vs*. 75.17 ± 16.51%) (Fig. 6) (S7, S8 and S9 Tables).

The EO was shown to be as effective as 0.2% CHX with regard to reducing the bacterial vitality after 4 days of rinsing (Fig. 5). In contrast, 0.2% CHX presented a higher activity when reducing the PL-Biofilm thickness (p<0.05) and the covering grade (p<0.001) after the same period of time (Figs. 4 and 6).

In relation to the bacterial vitality by layers, after the 4D-WATER and 4D-EO periods, the bacterial vitality was statistically higher (p<0.001) in the outer layers than in the inner ones. On the other hand, following the 4D-0.2% CHX period, there were no differences in terms of bacterial vitality among the 3 layers and although it was higher in outer layer, it did not reach significance (Table 1, intra-mouthwash analysis).

In regard to the layers comparison between the different periods, both EO and 0.2% CHX presented less bacterial vitality in all of them (Fig. 5) (S4 Table [see B] and S5 Table [see B]) in regard to the 4D-WATER period (S6 Table [see B]), with the differences being more marked between outer layers (p<0.001) and less pronounced (p<0.05) among the inner layers. There were no differences in terms of bacterial vitality among the 3 layers after the 4D-EO and 4D-0.2% CHX periods, being slightly higher in the outer layers in both cases (Table 1, inter-mouthwash analysis).

**Table 1:** Results derived from intra-mouthwash and inter-mouthwash comparisons on PL-biofilm bacterial vitality divided in layers.

PL-BIOFILM BACTERIAL VITALITY DIVIDED IN LAYERS			
INTRA-MOUTHWASH ANALYSIS			
	Layer 1 *vs*. Layer 2	Layer 1 *vs*. Layer 3	Layer 2 *vs*. Layer 3
4D-WATER	p<0.001	p<0.001	p<0.001
4D-EO	p<0.001	p<0.001	p<0.001
4D-0.2% CHX	—-	—-	—-
INTER-MOUTHWASH ANALYSIS			
	Layer 1 *vs*. Layer 1	Layer 2 *vs*. Layer 2	Layer 3 *vs*. Layer 3
4D-AE *vs*. 4D-WATER	p<0.001	p<0.001	p<0.05
4D-0.2% CHX *vs*.4D-WATER	p<0.001	p<0.001	p<0.05
4D-0.2% CHX *vs*.4D-AE	—-	—-	—-

4D-WATER = 4-day period during which rinses with 20 mL of sterile water are done twice a day; 4D-EO = 4-day period during which rinses with 20 mL of essential oils are done twice a day; 4D-0.2% CHX = 4-day period during which rinses with 10 mL of 0.2% chlorhexidine are done twice a day. Layer 1 = outer layer; Layer 2 = middle layer; Layer 3 = inner layer.

## Discussion

### Methodological approach

The design of a 4 day model was chosen because it measures the growth of the PL-Biofilm under the influence of a test solution, from a baseline of no dental plaque. If certain inhibition in the plaque formation cannot be demonstrated in this type of study, any significant effect must not be expected with a longer period of time [38].

There has been marked inter-individual variability detected regarding the characteristics of PL-Biofilm [11,13,14,33]. In the present study, after the calculation of the sample size, a group of 15 individuals was selected. This sample group is in the line of similar studies in which the number of volunteers ranged from 7 to 24 [10,13,14,28,29,39].

In regard to the vitality method selected, it has been stated that it was not possible to properly compare the vitality assessed with fluorescence staining solutions with traditional plaque cultures, because of the well-known limitations of the latter method (among others, only 50% of the oral bacteria are culturable [40]), which emphasises the necessity of using vitality assays [14,30]. The LIVE/DEAD BacLight fluorescence assay stains the bacteria in red or green depending on the permeability of their membrane. Given that the tested antiseptics act mostly at this cellular element, this vital staining method is suitable for this type of study.

Although there has been some discussion about the reliability of this technique [30,41], Hannig *et al*. [41] considered that live/dead staining methods were reliable when analysing antimicrobial agents activity, although they continued to ask the question about “how dead is dead?” due to several stages of vitality which have been discussed and described in the literature (viable and culturable, viable but non-culturable, dormant, non-viable and pre-lytic, and avital dead bacteria). The exact differentiation of these stages is still one of the greatest challenges in modern microbiology [42].

On the other hand, the BacLight system has recently been proposed as a reliable alternative when assessing bacterial vitality in natural dental biofilm [30]. Furthermore, the fact of doing 24 different measures (4 measures per disk; in total, 6 disks) in every volunteer in each of the tests, being at the same time, a crossover study, considerably reduce the potential bias that could exist by the determination of the bacterial vitality by this technique; in addition, it allows a better reproducibility in this type of studies.

Given that all microbiological techniques have their own disadvantages, and although the presented results are coherent with the clinical reality, the authors recognise the convenience of contrasting and complementing the data obtained with BacLight fluorescence solution with other molecular or bacteriological techniques or even with other fluorescent dyes.

### PL-Biofilm: thickness, bacterial vitality and covering grade after 4-day-rinsing periods with essential oils and and 0.2% chlorhexidine

Many short-term studies have been published which assess the effect of EO and CHX (among other antiseptics) on oral biofilm *in situ* [16,18,19,43]. The main disadvantage of this type of study is the fact that the dental plaque must be destructured if some parameters like bacterial vitality are to be measured, making it impossible to evaluate the architecture or actual thickness of the biofilm itself. This distortion would interfere in the delicate three-dimensional relationship existing between the bacteria inside the biofilm [11,12].

In contrast, there are fewer short-term studies on non-destructured PL-Biofilm evaluating antimicrobial agents like the CHX (analysed by TEM [39] or by CLSM [10,28,44], amine/stannous fluoride [10] or zinc chloride [29]. On the contrary, to the best of author’s knowledge, there are no published papers which compare the antiplaque effect of EO *vs*. 0.2% CHX in an *in situ* model of PL-Biofilm, analysing at the same time bacterial vitality, thickness and covering grade by that biofilm using CLSM techniques combined with fluorescence staining procedures.

In the present paper, the obtained thickness was 23.44 μm after 4-day-growing PL-Biofilm without any disturbing antiplaque agent. These results agree with those obtained by Jentsch *et al*., of 19.24 μm after 3 days [39], and Arweiler *et al*. of 25.33 μm after 5 days [28]. In contrast, they differ from those obtained by Gu *et al*. of 37 μm [29], and, to a greater extent, those from Auschill *et al*. of 76.7 μm [10], the latter 2 were after 2 days.

At this point, 2 issues should be commented upon; the first referring to the study of Jentsch *et al*. [39] in which they analysed a 3 day-evolution PL-Biofilm measured by TEM. This technique, despite being very precise, requires the fixation and drying of the PL-Biofilm, with unavoidable consequences to its delicate structure. The second is a very important methodological aspect which should be taken into account when using CLSM for measuring PL-Biofilm thicknesses and refers to the high thickness data obtained by Gu *et al*. [29] and mainly by Auschill *et al*. [10]. The method they used for thickness measurements is different from that used by other authors [28,37] and the current study. They determined the PL-Biofilm thickness using the number of 1-μm-planes obtained by CLSM (regardless of the perpendicularity); using a measurement like this could result in errors in many cases because a minimum substrate inclination could carry on an evaluation of the substrate in the diagonal, which would overestimate the thickness of the PL-Biofilm obtained. In this paper, the Leica Confocal SPII software was used to obtain the exact distance existing between the substrate and the highest point of the PL-Biofilm perpendicularly, which is a more realistic measure.

The thickness of the *in situ* PL-Biofilm after the 4-day-application of 0.2% CHX and EO was 6.48 μm and 9.99 μm, respectively. There are several studies [10,28,29,39,45] in which the PL-Biofilm thickness was analysed. The results differ between them depending on the technique used for its measurement (SEM, TEM or CLSM), type and concentration of the mouthwashes and its duration. The only study apart from the present series that compares the efficacy of EO and CHX is the one from Jentsch *et al*. [45] who measured the thickness of the PL-Biofilm developed on the surface of enamel slabs after a total period of 4 days. Unlike the findings reported in the present paper, they did not obtain any differences between the activity at reducing the PL-Biofilm thickness of the EO and the CHX. However, it should be noted that the concentration of CHX used in our study was higher (0.2%) than the used by Jentsch *et al*. [45]. Our results are similar to those obtained after the application of 0.2% CHX (8.6 μm after 2 days [10] and 11.91 μm after 5 days [28]. When a lower concentration was applied (0.12% CHX) the thickness rose to 14.02 μm after 3 days (TEM) [39] and 16.67 μm after 4 days (SEM) [45]. After the using of amine/stannous fluoride, the thicknesses obtained were 15.7 μm after 2 days [10], 11.91 μm after 3 days (TEM) and 13.25 μm after 4 days (SEM) [39]. Finally, after 2-day-use of zinc chloride at a concentration of 10 mM yielded a thickness of 10 μm [29].

In regard to the PL-Biofilm vitality, it was around the 56% after 4 days of growing in the present series. This contrasts with data obtained in other similar studies where the vitality was between 60 and 70% [10,29], but this was in a 2-day biofilm; these values are similar to those which the authors of this paper have obtained in previous studies on PL-Biofilm after 48 hours [13,14]. On the contrary, Arweiler *et al*. [28] obtained the same results (56.8%) in a PL-Biofilm which was, presumably, more similar to ours because of its 5-day-evolution. This lower vitality is highly influenced by the deepest layer of the PL-Biofilm (the nearest to the substrate), which is clearly where the differences are found in regard with the PL-Biofilm with less time of evolution. Arweiler *et al*. [28] also obtained a lower vitality in the deepest layer, coinciding with the theory that the bacteria located in the deepest part of a biofilm are in an inactive metabolic state [24,46].

With respect to the bacterial vitality after the mouthwash cycles with EO and 0.2% CHX, the bacterial vitality reductions are similar to those which have been previously obtained [10,28]. In the literature, for 0.2% CHX and amine/stannous fluoride, these values were 62% and 64%, respectively [10,28] and 43% for zinc chloride at a concentration of 10 mM [29]. In the present series, the bacterial vitality reduction was similar between both antiseptics (74% for EO and 77% for 0.2% CHX). Once again, the lowest bacterial vitality was found in the deepest layers of the PL-Biofilm.

In the present series, the results obtained both in thickness and bacterial vitality were considerably lower than those obtained by Arweiler *et al*. [28]. This could be because of some noticeable methodological differences that should be commented upon. In the present study, the volunteers did the negative and the test cycles in different periods, which permitted active mouthwashes being used *in situ* with the antiplaque agents. On the other hand, Arweiler *et al*. replaced the rinse with a simple immersion in the test solution (0.2 CHX). By doing this, they were assuming that an immersion was similar to an active mouthwash, ignoring the intrinsic factors that a rinse has itself such as the washing effect and the muscle strength applied by the cheeks. In a previous paper, Auschill *et al*. [10] highlighted the importance of doing the mouthwash *in situ* due to the fact that Pratten *et al*. [47] had previously exposed an *in vitro* biofilm to 0.2% CHX solution for 5 minutes, observing minimal effects on the biofilm. Based on this, in a previous pilot study, our research group observed that the effects of both 0.2% CHX and EO on the PL-Biofilm were not comparable at all in the case of an immersion and an active rinse (data unpublished).

The covering grade by the PL-Biofilm in combination with its thickness is directly related to the antiplaque capacity of an antiseptic agent. This parameter is important because it can be predictive of the adaptation of microorganisms to environmental influences [48]. To the best of author’s knowledge, there are no published papers on this issue in 4-day PL-Biofilm *in situ*. On the other hand, there are some *in vitro* studies on this topic and one of them obtained similar results to the present series after 4 days. It is one from Al-Ahmad *et al*. [49] who obtained a 77% and a 7% of covering grade with negative control and 0.2% CHX after 4 days, similar to those from the current study (75% and 20%, respectively).

There are two 4-day *in situ* studies which compare the effect of EO and CHX in terms of bacterial counts [17,50]. In these studies, the plaque control was done and culturable species were determined using culture plaque techniques. Eventually, they concluded that EO and 0.12% or 0.1% CHX (the concentrations used, respectively) had similar antiplaque effects. In the present series, although EO and 0.2% CHX presented a high and similar antibacterial activity, the latter was more powerful when inhibiting the PL-Biofilm formation, since both the covering grade and the thickness were considerably lower in its case.

Results obtained in microbiological studies, including the present paper, should be contrasted with those derived from clinical studies, based on the application of antiseptics in the short and long term. The results obtained in the present series are consistent with those from Riep *et al*. [15] and Pizzo *et al*. [43]. These 2 studies are clinical trials on the antiplaque activity of different antiseptics after 4 days, where both EO and CHX were effective when reducing the amount of dental plaque on the teeth surface in regard to the negative control after 4 days. In this sense, it should be mentioned that in multiple literature reviews it has been concluded that performing daily mouthwashes with EO produces a similar antiplaque effect to that of CHX [7,51,52]. On the contrary, in a relatively recent revision, Neely *et al*. [53] concluded that although the effect of the EO and the 0.2% CHX at reducing gingivitis may be equivalent, the latter is more effective when reducing the plaque formation after 6 months.

Although both the EO and the CHX has proved antiseptic efficacy on the PL-Biofilm, when used for long periods, some clinicians have to deal with possible side effects of both. In case of CHX, they are mainly dental staining [5] and taste alterations. When prescribing EO for long periods, there is always the controversy of using alcohol containing mouthwashes due to its possible (remains still unproven [54]) relation with the risk of developing oral cancer. For these reasons, a possible alternative could be the commercial formulas of EO without alcohol, that is why it would be interesting a new objective based on testing the EO without alcohol with this same model *in situ*, in order to evaluate its efficacy in the short term before testing it in the long term.

## Conclusion

In a 4-day *in situ* PL-Biofilm model, the formulas based on essential oils and 0.2% chlorhexidine showed a high antiplaque effect. Although 0.2% chlorhexidine formula showed better results when reducing the thickness and covering grade by the biofilm, both antiseptics showed a high and similar antibacterial activity. As a consequence, the present essential oils formula could be considered a reliable alternative to chlorhexidine in order to prevent its side effects when used continuously.

## Supporting Information

S1 CONSORT Checklist Consort 2010 Checklist of the present study. (DOC)

S1 Protocol Protocol of the study. A randomised, observer-masked, crossover study of the antiplaque efficacy of 2 available formulas based on EO and 0.2% CHX on an *in situ* model of PL-Biofilm growth.
(DOCX)

S1 Table Report of the primary data in terms of thickness of the biofilm obtained per disk and volunteer after 4D-EO. Results of thickness of the biofilm after 4D-EO.
(XLSX)

S2 Table Report of the primary data in terms of thickness of the biofilm obtained per disk and volunteer after 4D-CHX 0.2%. Results of thickness of the biofilm after 4D-CHX-0.2%.
(XLSX)

S3 Table Report of the primary data in terms of thickness of the biofilm obtained per disk andvolunteer after 4D-CONTROL. Results of thickness of the biofilm after 4D-CONTROL.
(XLSX)

S4 Table A. Report of the primary data in terms of bacterial vitality obtained per disk and volunteer after 4D-EO. Results of bacterial vitality of the biofilm after 4D-EO. **B.** Report of the primary data in terms of bacterial vitality obtained per layer, disk and volunteer after 4D-EO. Results of bacterial vitality of the biofilm after 4D-EO (BY LAYERS).
(XLSX)

S5 Table A. Report of the primary data in terms of bacterial vitality obtained per disk and volunteer after 4D-CHX 0.2%. Results of bacterial vitality of the biofilm after 4D- CHX 0.2%. **B.** Report of the primary data in terms of bacterial vitality obtained per layer, disk and volunteer after 4D-CHX 0.2%. Results of bacterial vitality of the biofilm after 4D- CHX 0.2% (BY LAYERS).
(XLSX)

S6 Table A. Report of the primary data in terms of bacterial vitality obtained per disk and volunteer after 4D-CONTROL. Results of bacterial vitality of the biofilm after 4D-CONTROL. **B.** Report of the primary data in terms of bacterial vitality obtained per layer, disk and volunteer after 4D-CONTROL. Results of bacterial vitality of the biofilm after 4D-CONTROL (BY LAYERS).
(XLSX)

S7 Table Report of the primary data in terms of covering grade of the biofilm obtained per disk and volunteer after 4D-EO. Results of covering grade of the biofilm after 4D-EO.
(XLSX)

S8 Table Report of the primary data in terms of covering grade of the biofilm obtained per disk and volunteer after 4D-CHX 0.2%. Results of covering grade of the biofilm after 4D-CHX 0.2%.
(XLSX)

S9 Table Report of the primary data in terms of covering grade of the biofilm obtained per disk and volunteer after 4D-CONTROL. Results of covering grade of the biofilm after 4D-CONTROL.
(XLSX)
