# Supplementary material for: Antiplaque Effect of Essential Oils and 0.2% Chlorhexidine on an In Situ Model of Oral Biofilm Growth: A Randomised Clinical Trial
Source: PLoS One. 2015 Feb 17;10(2):e0117177. doi: 10.1371/journal.pone.0117177 (PMC4331278; doi:10.1371/journal.pone.0117177)
Supplement: S1 Protocol — A randomised, observer-masked, crossover study of the antiplaque efficacy of 2 available formulas based on EO and 0.2% CHX on an in situ model of PL-Biofilm growth. (DOCX) [file pone.0117177.s002.docx]

**ANNEX I**

**ANALYSIS OF THE *IN SITU* ANTI-PLAQUE EFFECT OF ESSENTIAL OILS ON ORAL BIOFILM**

(DIRECTOR: INMACULADA TOMÁS CARMONA)

1. INTRODUCTION

The study of an antiseptic’s *in situ* antibacterial activity involves the analysis of its immediate effect and substantivity. Substantivity is defined as the antiseptic’s prolonged adherence to oral surfaces (teeth and mucous membranes) and its slow release at affective doses that guarantee the persistence of its antimicrobial activity (*Manau and Guasch, 2003*).

For several decades, the determination of salivary bacterial counts has been accepted by the scientific community to investigate the *in situ* antibacterial effect of antiseptics (*Addy and Moran, 1997; Sekino et al., 2003*), and has been considered a predictor of their substantivity and anti-plaque activity (*Roberts and Addy, 1981; Addy et al., 1989; Jenkins et al., 1990; Jenkins et al., 1994; Fine et al., 2000; da Silva et al., 2005; Otten et al., 2010)*. After the first results published by Schiött et al. in 1970 (*Schiött et al., 1970*), there have been many published studies that have evaluated the substantivity of different antiseptics, such as CHX and essential oils, on salivary flora (*Moran et al., 1992; Jenkins et al., 1994; Elworthy et al., 1996; Balbuena et al., 1998; Fine et al., 2000; da Silva et al., 2005; Otten et al., 2010*). In most of the published series, the *in vivo* antimicrobial activity of antiseptics in saliva was determined using microbiological culture techniques (*Moran et al., 1992; Jenkins et al., 1994; Elworthy et al., 1996; Balbuena et al., 1998; Tomás et al., 2008; Fine et al., 2000; Otten et al., 2010*). Some authors, however, questioned the reliability of these techniques and proposed the use of fluorescence methods that use specific fluorochromes to label viable and non-viable bacteria (*Weiger et al., 1998; Caballero et al., 2009; Tomás et al., 2009*).

*In vitro* experiments have shown that dental biofilm has greater resistance to antibacterial agents as the result of a slow growth rate, the difficulty involved in penetrating its structure and possible inactivation of the agent inside it (*Wood et al., 2000*). Therefore, the other oral ecological niche in which the *in situ* antibacterial activity of antiseptics has been widely studied is bacterial plaque (*Fine et al., 2000; Pan et al., 2000;* *König et al., 2002; Arweiler et al., 2002; van der Mei et al., 2006; García-Caballero et al., 2008; Otten et al., 2010*). In order to improve the methodology used in such studies, some authors designed special removable apparatuses containing different disks on which dental biofilm grew (*Netuschil et al., 1998; Auschill et al., 2001; Arweiler et al., 2004; Auschill et al., 2005; García-Caballero et al., 2008*). Subsequently, these “non-destructured” bacterial plaques were analysed by confocal laser microscope techniques and fluorescent solutions that enabled the simultaneous study of the three-dimensional structure of biofilm and the assessment of bacterial vitality (*Netuschil et al., 1998; Auschill et al., 2001; Arweiler et al., 2004; Auschill et al., 2005; García-Caballero et al., 2008*). However, few published studies have evaluated the antibacterial efficacy of essential oils on dental biofilm *in situ* (*Pan et al., 2000*).

1. OBJECTIVES

Having previously developed a first objective based on the assessment of the *in situ* substantivity of a single mouthwash with essential oils on “non-destructured” bacterial plaque, we now contemplate a second objective, consisting of assessing the *in situ* anti-plaque effect of several essential oil mouthwashes on “non-destructured” bacterial plaque in a 4-day growth model.

The methodology will involve the application of epifluorescence microscopy and confocal laser microscopy techniques and *LIVE/DEAD^®^ BacLight™* double-staining solution.

1. **MATERIAL, METHODS AND WORK PLAN**

This is a randomized, observed-masked, crossover study of the antibacterial efficacy of essential oils on an *in situ* model of plaque like-biofilm growth.

- Sample size and study group selection

Assuming these criteria and the possible application of repeated measures ANOVA test, a sample size of 15 subjects was required. The sample size calculation was performed using the program G*Power 3.1.5. The study group will be formed of 15 systemically healthy adult volunteers.

- Inclusion Criterea:
- Age: between 20 and 45 years old.
- Good oral health status:
  1. Minimum of 24 permanent teeth excluding from the tooth count:
     - The third molars.
     - Teeth extensively restored.
     - Abutments.
     - Teeth exhibiting severe generalized cervical and/or enamel abrasion.
  2. No evidence of gingivitis or periodontitis (Community Periodontal Index score = 0) *(World Health Organisation, 1997).*
  3. Absence of caries.
- Exclusion Criteria:
  - Smoker.
  - Presence of dental prostheses or orthodontic devices
  - Routine use of oral antiseptic during the previous 3 months.
  - Presence of any systemic disease that could alter the production or composition of the saliva.
  - Self-reported pregnancy or lactation.

Previously to the start of this study, a professional toothcleaning will be performed to all volunteers.

- **Construction of the disk-holding apparatus**

After considering a number of previously described *in situ* models (*Auschill et al., 2004; Arweiler et al., 2004*), an individualized splint of the lower arch was created for each volunteer, able to hold six glass disks (6 mm in diameter, 1 mm thickness) and polished at 800 grit. The characteristics of this splint have been previously described by other (*Tomás et al., 2010*), although a new variety of splint has now been introduced; the old one was a complete individual inferior arch splint, which has now been broken in two, going from the last molar to the homolateral canine.

- **Application of the antimicrobial agents**

During 96 hours (4 days), each volunteer will wear the splints with the glass disks, removing them only for eating and oral hygiene measures (exclusively based on mechanical measures -toothbrush without any type of toothpaste-); during the time the splint is not in the mouth it will be kept inside an opaque container in humidity conditions which will be provided to each one of the volunteers. The time limit that the splints can be out of the mouth has been estimated in 20 minutes, so the volunteers will be asked to eat in the minimum time possible. Two daily mouthwashes will be done each of the 4 days, in the morning and in the late-evening with the splints inside their mouth, after having done the corresponding oral mechanic hygiene measures.

- Plaque like-biofilm recollection

The sample recollection will be done individually and at first hour in the morning. Each day we will work only with a volunteer. The disks will be removed from the splints and they will be analyzed.

After the 4-day period wearing the splints, each volunteer will have done:

1) Mouthwashes with 20 ml for 30 seconds with essential oils/twice a day (Listerine Mentol, Listerine^®^, Johnson & Johnson, Madrid, Spain).

2) Mouthwashes with 10 mL for 30 seconds with chlorhexidine 0.2%/twice a day (Oraldine Perio^®^, Johnson & Johnson, Madrid, Spain) (positive control).

3) Mouthwashes with 20 mL for 30 seconds with sterile water/twice a day (negative control).

Applying a balanced randomisation system, all the volunteers will perform the 3 rinse cycles, establishing a 2 week “washout period” between the different applications.

**1**

- Preparation of the fluorescent solution

The fluorescent solution *LIVE/DEAD^®^ BacLight™ (Molecular Probes, Leiden, The Netherlands)* comprises 2 fluorochromes, SYTO 9 and propidium iodide (PI), which when applied simultaneously, enable us to distinguish between bacteria with intact membranes (emit green fluorescence) and bacteria with damaged membranes (emit red fluorescence).

In 4 ml of sterile water and filtered through a Millipore membrane filter (Millipore Ibérica S.A., Madrid, Spain), reaching a 1:1 proportion of the two fluorochromes, and will be stored at -20ºC. The excitation wave lengths (range) for SYTO 9 and PI are 488 nm (480-500 nm) and 561 nm (490-635 nm), respectively.

- Bacterial plaque sample processing

The glass disks will be removed from the apparatus and immediately submerged in 100 μl of the fluorescent solution, and stored in a dark chamber at room temperature for 15 minutes. They will be observed under a microscope by an investigator not conversant with the study design, using a *Laser Confocal Espectral Leica TCS SP2* microscope (Leica Microsystems Heidelberg GmbH, Mannheim, Germany) and the HCX APO L 63x/0.9 lens for underwater use. Biofilm thickness (BT, mm), area occupied by biofilm (AB, %) and   bacterial vitality (BV, %) will be evaluated.

In the central part of each disk, 4 randomly selected fields or XYZ series in the central part of each disk will be evaluated. Fluorescence emission will be determined in series of XY images in which each image corresponded with each one of the Z positions (depth). The optical sections will be scanned in one micron sections from the surface of the biofilm to its base, measuring the maximum thickness of the field and subsequently the mean thickness of the biofilm of the corresponding sample. The biofilm maximum thickness of each field will be divided into 3 zones or layers equivalent: inner layer (layer 3), middle layer (layer 2) and outer layer (layer 1). After obtaining the bacterial vitality (total and layer by layer) and the biofilm thickness of the 4 fields in each disk, an average result will be obtained for each variable on every volunteer. These results will be compared to the positive and negative controls.

Bacterial vitality in series of xy images will be quantified by cytofluorogram analysis (*Leica Confocal Software*). In this analysis, the images of each fluorochrome are described as “channels” (SYTO 9 is in the “green channel” and PI in the “red channel”), obtaining the area (µm^2^) occupied by each channel, the total area occupied by biofilm and the respective vitality percentage.

- Statistical analysis

The results will be analysed with the PASW***^®^*** statistical program, version 20 for Windows (*SPSS Inc., Chicago, USA*). ANOVA with repeated measures will be used for inter-layer comparisons of biofilm at baseline and inter-antimicrobial comparisons. Pairwise comparisons (with Bonferroni adjustment) will be used for the inter-layer and inter-antimicrobial analysis between 2 biofilm samples. Statistical significance will be a P value less than 0.05.

1. **BIBLIOGRAPHY**

Addy M, Jenkins S, Newcombe R. Toothpastes containing 0.3% and 0.5% triclosan. II. Effects of single brushings on salivary bacterial counts. *Am J Dent* 1989; 2: 215-219.

Addy M, Moran J. Evaluation of oral hygiene products: science is true; don’t be misled by the facts. *Periodontology 2000* 1997; 15: 40-51.

Arweiler NB, Hellwig E, Sculean A, Hein N, Auschill TM. Individual vitality pattern of *in situ* dental biofilms at different locations in the oral cavity. *Caries Res* 2004; 38: 442-447.

Auschill TM, Artweiler NB, Netuschil L, Brecx M, Reich E, Sculean A. Spatial distribution of vital and dead microorganisms in dental biofilms. *Arch Oral Biol* 2001; 46: 471-476.

Auschill TM, Hein N, Hellwig E, Follo M, Sculean A, Arweiler N.B. Effect of two antimicrobial agents on early *in situ* biofilm formation. *J Clin Periodontol* 2005; 32: 147-152.

Auschill TM, Hellwig E, Sculean A, Hein N, Arweiller NB. Impact of the intraoral location on the rate of biofilm growth. *Clin Oral Invest* 2004; 8: 97-101.

Balbuena L, Stambaugh KI, Ramirez SG, Yeager C. Effects of topical oral antiseptic rinses on bacterial counts of saliva in healthy human subjects. *Otolaryngol Head Neck Surg* 1998; 118: 625-629.

García-Caballero L, Tomás Carmona I, Cousido M, Limeres J, Torres S, Diz P. Substantivity of a chlorhexidine mouthrinse on saliva and biofilm. PEF International Association for Dental Research. Londres, 2008.

Da Silva CM, Colombo AV, do Souto RM, Colombo AP. *In vivo* evaluation of the effect of essential oil-containing oral strips on salivary bacteria using the checkerboard method. *J Clin Dent* 2005; 16: 38-43.

Elworthy A, Greenman J, Doherty FM, Newcombe RG, Addy M. The substantivity of a number of oral hygiene products determined by the duration of effects on salivary bacteria. *J Periodontol* 1996; 67: 572-576.

Fine DH, Furgang D, Barnett ML, Drew C, Steinberg L, Charles CH, Vincent JW. Effect of an essential oil-containing antiseptic mouthrinse on plaque and salivary Streptococcus mutans levels. *J Clin Periodontol* 2000; 27: 157-61.

García-Caballero L, Tomás Carmona I, Cousido M, Limeres J, Torres S, Diz P. Substantivity of a chlorhexidine mouthrinse on saliva and biofilm. PEF International Association for Dental Research. Londres, 2008.

Jenkins S, Addy M, Newcombe R. The effects of 0.5% chlorhexidine and 0.2% triclosan containing toothpastes on salivary bacterial counts. *J Clin Periodontol* 1990; 17: 85-89.

Jenkins S, Addy M, Wade W, Newcombe RG. The magnitude and duration of the effects of some mouthrinse products on salivary bacterial counts. *J Clin Periodontol* 1994; 21: 397-401.

König J, Storcks V, Kocher T, Bössmann K, Plagmann HC. Anti-plaque effect of tempered 0.2% chlorhexidine rinse: an *in situ* study. *J Clin Periodontol* 2002; 29: 207-210.

Manau Navarro C, Guasch Serra S. Métodos de control de la placa bacteriana. En: Cuenca Sala E, Manau Navarro C, Serra Majem L (eds). Odontología preventiva y comunitaria: Principios, métodos y aplicaciones, 2a edn. Masson: Barcelona. 2003: 79.

Moran J, Addy M, Wade WG, Maynard JH, Roberts SE, Aström M, Movert R. A comparison of delmopinol and chlorhexidine on plaque regrowth over a 4-day period and salivary bacterial counts. *J Clin Periodontol* 1992; 19: 749-753.

Netuschil L, Reich E, Unteregger G, Sculean A, Brecx M. A pilot study of confocal laser scanning microscopy for the assessment of undisturbed dental plaque vitality and topography. *Arch Oral Biol* 1998; 43: 277-285.

Otten MP, Busscher HJ, vander Mei HC, Abbas F, van Hoogmoed CG. Retention of antimicrobial activity in plaque and saliva following mouthrinse use *in vivo*. *Caries Res* 2010; 44: 459-64.

Pan P, Barnett M.L, Coelho J, Brogdon C, Finnegan M.B. Determination of the *in situ* bactericidad activity o fan Essentials oil mouthrinse using a vital stain method. *J Clin Periodontol* 2000; 27: 256

Roberts WR, Addy M. Comparison of the *in vivo* and *in vitro* antibacterial properties of antiseptic mouthrinses containing chlorhexidine, alexidine, cetyl pyridinium chloride and hexetidine. Relevance to mode of action. *J Clin Periodontol* 1981; 8: 295-310.

Schiött CR, Loe H, Jensen SB, Kilian M, Davies RM, Glavind K. The effect of chlorhexidine mouthrinses on the human oral flora. *J Periodontal Res* 1970; 5: 84-89.

Sekino S, Ramberg P, Uzel NG, Socransky S, Lindhe J. Effect of various chlorhexidine regimens on salivary bacteria and *de novo* plaque formation. *J Clin Periodontol* 2003; 30: 919-925.

Tomás Carmona I, Cousido MC, Tomás M, Limeres J, García-Caballero L, Diz P. *In vivo* bactericidal effect of 0.2% chlorhexidine but not 0.12% on salivary obligate anaerobes. *Arch Oral Biol* 2008; 53: 1186-1191.

Tomás Carmona I, García-Caballero L, Cousido M, Limeres J, Álvarez M, Diz P. Evaluation of chlorhexidine substantivity on salivary flora by epifluorescence microscopy. *Oral Dis* 2009; 15: 428-33.

Tomás I HB, Diz P, Donos N. In vivo oral biofilm analysis by confocal laser scanning microscopy: methodological approaches. In: A M-V (ed) Microscopy. Science, technology, applications and education. 2010. Formatex, Badajoz (Spain), pp 597-606.

van der Mei HC, White DJ, Atema-Smit J, van de Belt-Gritter E, Busscher HJ. A method to study sustained antimicrobial activity of rinse and dentifrice components on biofilm viability *in situ*. *J Clin Periodontol* 2006; 33: 14-20.

Weiger R, Netuschil L, Wester-Ebbinghaus T, Brecx M. An approach to differentiate between antibacterial and antiadhesive effects of mouthrinses *in vivo*. *Arch Oral Biol* 1998; 43: 559-565.

Wood SR, Kirkham J, Marsh PD, Shore RC, Nattress B, Robinson C. Architecture of intact natural human plaque biofilms studied by confocal laser scanning microscopy. *J Dent Res* 2000; 79: 21-27.

World Health Organisation. Oral health surveys, Basic methods, 4th ed. WHO: Geneva, 1997.
